# Bis(4-sulfamoylanilinium) sulfate

**DOI:** 10.1107/S1600536813007216

**Published:** 2013-03-28

**Authors:** B. Ravikumar, S. Pandiarajan, S. Athimoolam

**Affiliations:** a Department of Physics, Devanga Arts College, Aruppukottai 626 101, India; b Department of Physics, University College of Engineering Nagercoil, Anna University Chennai, Nagercoil 629 004, India

## Abstract

In the title salt, 2C_6_H_9_N_2_O_2_S^+^·SO_4_^2−^, the sulfate S atom is situated on a crystallographic twofold axis (the symmetry of the anion is 2). The anion exerts intense libration, which is manifested by shortening of the observed sulfate S—O bonds, as well as by features in the electron-density map. The crystal structure is stabilized through a three-dimensional hydrogen-bonding network formed by strong N—H⋯O hydrogen bonds.

## Related literature

For information about folate synthesis, see: Kent (2000 ▶). For related structures, see: Pandiarajan *et al.* (2011 ▶); Zaouali Zgolli *et al.* (2010 ▶). For correction of the S—O distances in the sulfate anion due to libration movement, see: Nardelli (1995 ▶). For TLS approximation, see: Schomaker & Trueblood (1968 ▶). For graph-set motifs, see: Etter *et al.* (1990 ▶). For the categorization of hydrogen bonds, see: Desiraju & Steiner (1999 ▶).
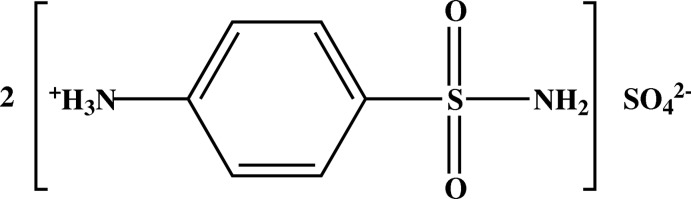


## Experimental

### 

#### Crystal data

2C_6_H_9_N_2_O_2_S^+^·SO_4_^2−^
*M* *_r_* = 442.48
Orthorhombic, 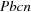

*a* = 9.6543 (6) Å
*b* = 9.7591 (11) Å
*c* = 18.579 (3) Å
*V* = 1750.5 (4) Å^3^
*Z* = 4
Mo *K*α radiation
μ = 0.48 mm^−1^
*T* = 293 K
0.24 × 0.22 × 0.19 mm

#### Data collection

Bruker SMART APEX CCD area-detector diffractometer
18054 measured reflections
2054 independent reflections
1950 reflections with *I* > 2σ(*I*)
*R*_int_ = 0.025

#### Refinement

*R*[*F* ^2^ > 2σ(*F* ^2^)] = 0.039
*wR*(*F* ^2^) = 0.104
*S* = 1.05
2054 reflections
131 parameters
2 restraints
H atoms treated by a mixture of independent and constrained refinement
Δρ_max_ = 0.69 e Å^−3^
Δρ_min_ = −0.51 e Å^−3^

### 

Data collection: *SMART* (Bruker, 2001 ▶); cell refinement: *SAINT* (Bruker, 2001 ▶); data reduction: *SAINT*; program(s) used to solve structure: *SHELXTL/PC* (Sheldrick, 2008 ▶); program(s) used to refine structure: *SHELXTL/PC* and *JANA2006* (Petricek *et al.*, 2006 ▶); molecular graphics: *PLATON* (Spek, 2009 ▶) and *JANA2006*; software used to prepare material for publication: *SHELXTL/PC*.

## Supplementary Material

Crystal structure: contains datablock(s) global, I. DOI: 10.1107/S1600536813007216/fb2278sup1.cif

Structure factors: contains datablock(s) I. DOI: 10.1107/S1600536813007216/fb2278Isup2.hkl

Supplementary material file. DOI: 10.1107/S1600536813007216/fb2278Isup3.cml

Additional supplementary materials:  crystallographic information; 3D view; checkCIF report

## Figures and Tables

**Table 1:** Hydrogen-bond geometry (Å, °)

*D*—H⋯*A*	*D*—H	H⋯*A*	*D*⋯*A*	*D*—H⋯*A*
N1—H1*N*⋯O22^i^	0.84 (2)	1.93 (2)	2.743 (3)	164 (3)
N1—H2*N*⋯O21^ii^	0.86 (2)	1.99 (2)	2.849 (3)	168 (3)
N2—H2*B*⋯O2^iii^	0.89	2.30	3.043 (3)	141
N2—H2*B*⋯O1^iv^	0.89	2.57	3.146 (2)	123
N2—H2*A*⋯O22^v^	0.89	1.90	2.787 (3)	177
N2—H2*C*⋯O21^vi^	0.89	2.06	2.900 (3)	158

Symmetry codes: (i) 
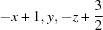
; (ii) 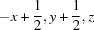; (iii) 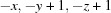; (iv) 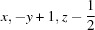; (v) 
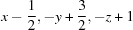
; (vi) 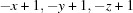.

**Fig. 1.**
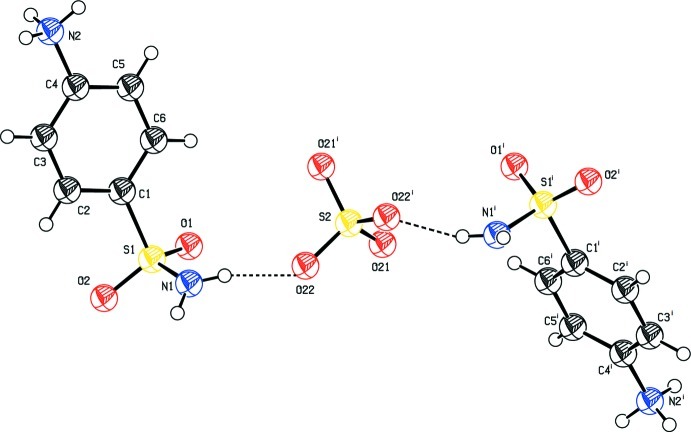
The title molecule with the atom numbering scheme. The displacement ellipsoids are shown at the 50% probability level. The H-bonds are shown as dashed lines. (Symmetry code: (i) 1-x, y, 3/2-z.)

**Fig. 2.**
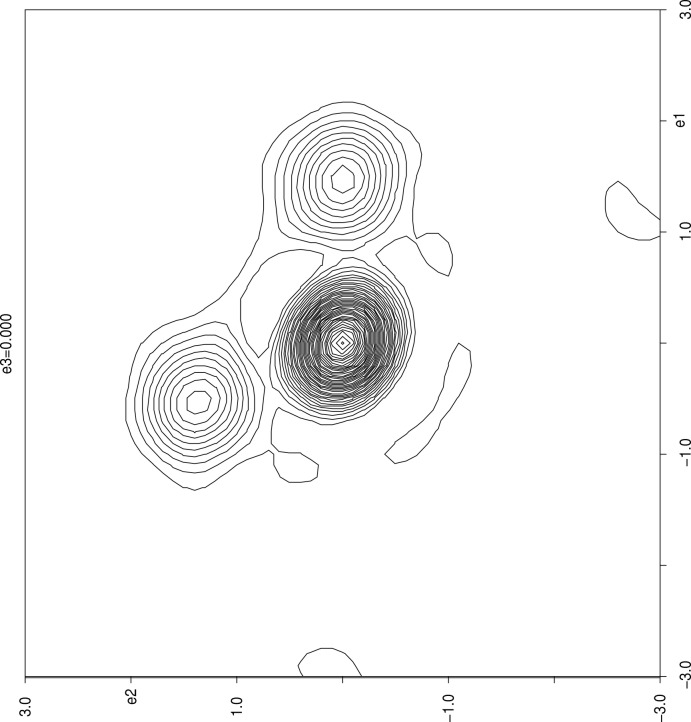
Section through the electron density map calculated from Fo passing through the atoms S2 and the atoms O21 and O21i (Petricek et al., 2006). Symmetry code: (i) 1-x, y, 3/2-z. The contours are given in 1 eÅ-3 levels.

**Fig. 3.**
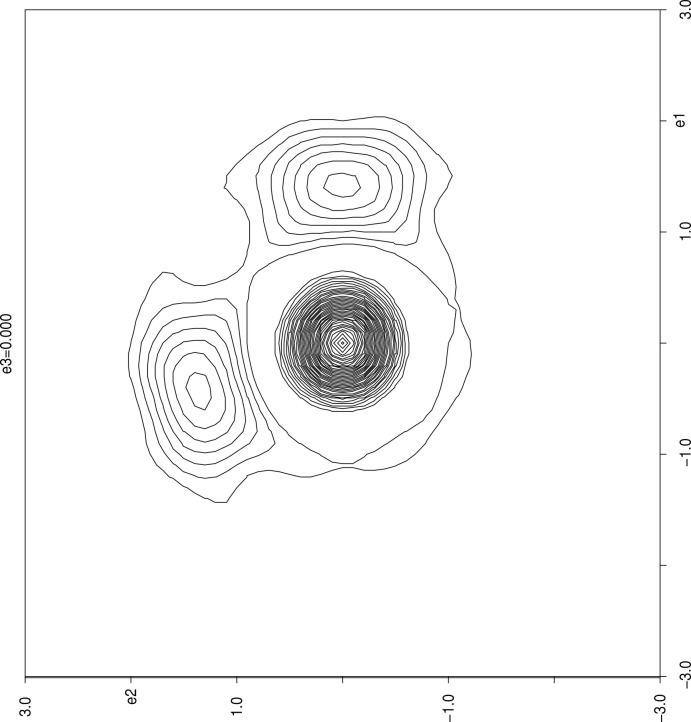
Section through the electron density map calculated from Fo passing through the atoms S2 and the atoms O22 and O22i (Petricek et al., 2006). Symmetry code: (i) 1-x, y, 3/2-z. The contours are given in 1 eÅ-3 levels. The curved character of the electron density of the depicted O atoms indicates libration movement.

**Fig. 4.**
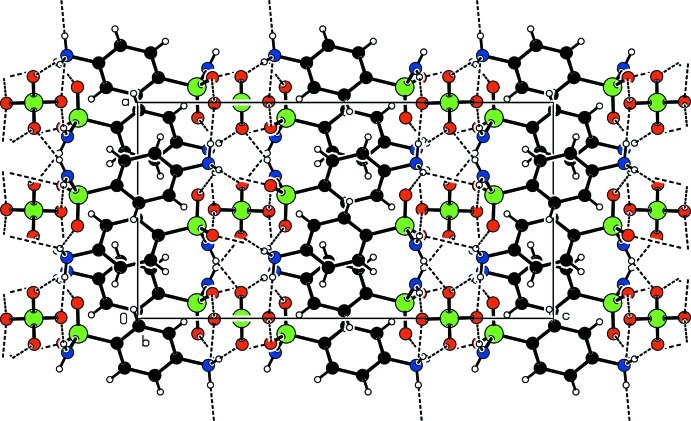
Packing diagram of the title compound viewed down the b axis. The H-bonds are shown as dashed lines.

## supplementary crystallographic information

### Comment

Sulfonamides have been the first antibacterial drugs and paved the way for the antibiotic revolution in medicine. Sulfanilamides, which belong to sulfonamide drugs, act as competitive inhibitors for the enzyme dihydropteroate synthetase (DHPS). This enzyme is involved in the folate synthesis (Kent, 2000).

We have recently reported a nitrate complex of sulfanilamide (Pandiarajan *et al.*, 2011). In continuation of our interest on the sulfanilamide complexes, the synthesis of the title compound and its title structure, bis(4-sulfamoylanilinium) sulfate, is described here.

In the title structure, the sulfate anion is situated in the special position on the twofold axis, thus the symmetry of this molecule is 2. The refinement has shown that the displacement ellipsoid of the sulfate atom O22 is extensively elongated while that of O21 has shown usual behaviour (Fig. 1). This is due to the libration movement as it is indicated by Figs. 2 and 3 which show the sections of the electron density maps (Petricek *et al.*, 2006) through S2 and the atoms O21 and O22, respectively. Note the curved electron density pertinent to O22 (Fig. 3). The libration movement is also manifested by the values of the corrected S—O distances (Nardelli, 1995): The uncorrected values S2—O21 and S2—O22 are 1.475 (2) and 1.436 (2) Å, respectively, while the respective corrected values equal to 1.501 and 1.516 Å. The attempts to express the libration of the sulfate by TLS (Schomaker & Trueblood, 1968; *JANA2006* (Petricek *et al.*, 2006) failed in this case.

The geometric parameters of the cation in the title structure is in agreement with the reported structures of 4-sulfamoylanilinium nitrate (Pandiarajan *et al.*, 2011) and 4-sulfamoylanilinium chloride (Zaouali Zgolli *et al.*, 2010).

The crystal structure is stabilized through a three-dimensional hydrogen bonding network formed by strong N—H···O hydrogen bonds (Table 1, Fig. 4). (The nomenclature regarding the strength of the hydrogen bonds was taken from Desiraju & Steiner, 1999.) The sulfate oxygens as well as the oxygen O2 of the sulfonamyl group are the acceptors of these strong hydrogen bonds (Table 1) which are donated by the primary amine as well as by the ammonium groups. Among the N—H···O hydrogen bonds, one of the N—H···O hydrogen bonds is observed to be a bifurcated hydrogen bond, with one donor hydrogen (Table 1): N2—H2B···O2 (-*x*, -*y*+1, -*z*) and N2—H2B···O1 (*x*, -*y*+1, -*z*+1/2). All other hydrogen bonding interactions are cation-anion type. These hydrogen bonds are localized in layers parallel to (0 0 1) which are situated at *z* = 1/4 and *z* = 3/4. Hence, hydrophilic and hydrophobic regions alternate along *c* axis as a result of the arrangement of anions and the aromatic cationic parts.

Among the important graph set motifs pertinent to the hydrogen bonding in the structure can be named *R*_2_^2^(16) (Etter *et al.*, 1990): The cationic –NH_3_ group is bonded to the O atom of the S═O group of another cation through the N2—H2B···O2^iii^ hydrogen bond (the symmetry code iii: -*x*, 1-*y*, 1-*z*). These bonds are involved in a ring motif *R*_2_^2^(16) about the crystallographic inversion centre situated at the Wyckoff position 4*a*.

Among other graph-set motifs which can be discerned in this hydrogen bond pattern, *C*_4_^4^(12) is worthwhile mentioning. This motif contains N2—H2A···O22^v^—S2^v^— O21^v^···H2N^vii^-N1^vii^-H1N^vii^···O22^viii^-S2^viii^-O21^viii^··· H2C^ix^-N2^ix^··· where the symmetry codes are v : -1/2+*x*, 3/2-*y*, 1-*z*; vii : -*x*, 2-*y*, 1-*z*; viii : 1-*x*, 2-*y*, -1/2+*z*; ix : -*x*, 1+*y*, 1/2-*z*.

### Experimental

The synthesis of the title compound was carried out by heating of the mixture of sulphanilamide (3.4 g) and sulfuric acid (0.5 ml of 98% concentration) in 20 ml of water as the stoichiometric ratio of 2:1 (at 60°C) under reflux for 1 h. Colourless prismatic crystals of bis(4-sulfamoylanilinium) sulfate suitable for single-crystal X-ray analysis with the approximate size of 1.6 cm × 0.8 cm × 0.3 cm were obtained by slow evaporation at room temperature. The measured sample was cut from a bigger crystal.

### Refinement

All the H atoms were discernible in the difference electron density map. Nevertheless, the aryl H atoms were constrained and refined in the riding atom approximation: C_aryl_—H_aryl_ = 0.93 Å and *U*_iso_(H_aryl_)= 1.2*U*_eq_(C_aryl_). The other atoms are involved in the N—H···O hydrogen bonds and after some tests also the ammonium H atoms were constrained in idealized tetrahedral symmetry because a distance-restrained refinement yielded improbable differences in N2—H2A, N2—H2B, N2—H2C distances. The pertinent constraints were N2—H_ammonium_ = 0.89 Å and *U*_iso_(H_ammonium_)= 1.5*U*_eq_(N_ammonium_). The distances of the primary amine H atoms to their carrier atom N1 were restrained to 0.86 (1) Å while the *U*_iso_(H_amine_)= 1.2*U*_eq_(N_amine_).

### Crystal data

**Table:**

2C_6_H_9_N_2_O_2_S^+^·SO_4_^2^^−^	*F*(000) = 920
*M**_r_* = 442.48	*D*_x_ = 1.679 Mg m^−^^3^
Orthorhombic, *P**b**c**n*	Mo *K*α radiation, λ = 0.71073 Å
Hall symbol: -P 2n 2ab	Cell parameters from 2417 reflections
*a* = 9.6543 (6) Å	θ = 2.4–23.9°
*b* = 9.7591 (11) Å	µ = 0.48 mm^−^^1^
*c* = 18.579 (3) Å	*T* = 293 K
*V* = 1750.5 (4) Å^3^	Block, colourless
*Z* = 4	0.24 × 0.22 × 0.19 mm

### Data collection

**Table:**

Bruker SMART APEX CCD area-detector diffractometer	1950 reflections with *I* > 2σ(*I*)
Radiation source: fine-focus sealed tube	*R*_int_ = 0.025
Graphite monochromator	θ_max_ = 27.8°, θ_min_ = 2.2°
ω scans	*h* = −12→12
18054 measured reflections	*k* = −12→12
2054 independent reflections	*l* = −24→23

### Refinement

**Table:**

Refinement on *F*^2^	Secondary atom site location: difference Fourier map
Least-squares matrix: full	Hydrogen site location: difference Fourier map
*R*[*F*^2^ > 2σ(*F*^2^)] = 0.039	H atoms treated by a mixture of independent and constrained refinement
*wR*(*F*^2^) = 0.104	*w* = 1/[σ^2^(*F*_o_^2^) + (0.0478*P*)^2^ + 1.7836*P*] where *P* = (*F*_o_^2^ + 2*F*_c_^2^)/3
*S* = 1.05	(Δ/σ)_max_ = 0.001
2054 reflections	Δρ_max_ = 0.69 e Å^−^^3^
131 parameters	Δρ_min_ = −0.51 e Å^−^^3^
2 restraints	Extinction correction: *SHELXTL/PC* (Sheldrick, 2008), Fc^*^=kFc[1+0.001xFc^2^λ^3^/sin(2θ)]^-1/4^
29 constraints	Extinction coefficient: 0.048 (2)
Primary atom site location: structure-invariant direct methods	

### Special details

**Table:**

Geometry. All esds (except the esd in the dihedral angle between two l.s. planes) are estimated using the full covariance matrix. The cell esds are taken into account individually in the estimation of esds in distances, angles and torsion angles; correlations between esds in cell parameters are only used when they are defined by crystal symmetry. An approximate (isotropic) treatment of cell esds is used for estimating esds involving l.s. planes.
Refinement. Refinement of *F*^2^ against ALL reflections. The weighted *R*-factor *wR* and goodness of fit *S* are based on *F*^2^, conventional *R*-factors *R* are based on *F*, with *F* set to zero for negative *F*^2^. The threshold expression of *F*^2^ > σ(*F*^2^) is used only for calculating *R*-factors(gt) *etc*. and is not relevant to the choice of reflections for refinement. *R*-factors based on *F*^2^ are statistically about twice as large as those based on *F*, and *R*- factors based on ALL data will be even larger.

### Fractional atomic coordinates and isotropic or equivalent isotropic displacement parameters (Å^2^)

**Table:**

	*x*	*y*	*z*	*U*_iso_*/*U*_eq_	
C1	0.11530 (19)	0.63165 (19)	0.54983 (9)	0.0275 (4)	
C2	0.0350 (2)	0.6931 (2)	0.49691 (11)	0.0346 (4)	
H2	−0.0419	0.7451	0.5098	0.041*	
C3	0.0693 (2)	0.6772 (2)	0.42437 (10)	0.0349 (4)	
H3	0.0161	0.7185	0.3887	0.042*	
C4	0.18330 (18)	0.59911 (18)	0.40639 (9)	0.0278 (4)	
C5	0.2642 (2)	0.5368 (2)	0.45866 (10)	0.0363 (4)	
H5	0.3407	0.4846	0.4455	0.044*	
C6	0.2300 (2)	0.5530 (2)	0.53135 (10)	0.0368 (4)	
H6	0.2833	0.5115	0.5669	0.044*	
N1	0.1578 (2)	0.7855 (2)	0.66870 (10)	0.0418 (4)	
H1N	0.234 (2)	0.771 (3)	0.6883 (13)	0.050*	
H2N	0.118 (3)	0.863 (2)	0.6780 (15)	0.050*	
N2	0.21752 (18)	0.57958 (18)	0.32972 (8)	0.0343 (4)	
H2A	0.1882	0.6516	0.3046	0.052*	
H2B	0.1762	0.5041	0.3135	0.052*	
H2C	0.3088	0.5712	0.3248	0.052*	
S1	0.07157 (5)	0.65527 (5)	0.64256 (2)	0.02962 (17)	
O1	0.11508 (19)	0.53240 (16)	0.68009 (8)	0.0447 (4)	
O2	−0.07144 (14)	0.69235 (18)	0.64486 (8)	0.0401 (4)	
S2	0.5000	0.61321 (7)	0.7500	0.0330 (2)	
O21	0.5039 (2)	0.52764 (18)	0.68459 (10)	0.0586 (5)	
O22	0.6198 (3)	0.7004 (3)	0.75120 (13)	0.1099 (12)	

### Atomic displacement parameters (Å^2^)

**Table:**

	*U*^11^	*U*^22^	*U*^33^	*U*^12^	*U*^13^	*U*^23^
C1	0.0286 (8)	0.0291 (8)	0.0249 (8)	−0.0019 (7)	0.0024 (7)	−0.0003 (6)
C2	0.0336 (9)	0.0373 (10)	0.0328 (9)	0.0103 (8)	0.0029 (8)	0.0007 (8)
C3	0.0359 (10)	0.0398 (10)	0.0291 (9)	0.0076 (8)	−0.0016 (7)	0.0055 (8)
C4	0.0298 (8)	0.0287 (8)	0.0249 (8)	−0.0040 (7)	0.0036 (6)	−0.0001 (6)
C5	0.0325 (9)	0.0433 (11)	0.0330 (9)	0.0102 (8)	0.0046 (8)	0.0015 (8)
C6	0.0343 (10)	0.0469 (11)	0.0293 (9)	0.0109 (8)	0.0000 (7)	0.0046 (8)
N1	0.0381 (9)	0.0407 (10)	0.0465 (10)	−0.0023 (8)	−0.0103 (8)	−0.0076 (8)
N2	0.0371 (9)	0.0398 (9)	0.0261 (7)	−0.0027 (7)	0.0044 (6)	−0.0013 (6)
S1	0.0324 (3)	0.0309 (3)	0.0255 (2)	−0.00172 (17)	0.00340 (16)	−0.00089 (16)
O1	0.0638 (10)	0.0385 (8)	0.0317 (7)	0.0043 (7)	0.0071 (7)	0.0072 (6)
O2	0.0300 (7)	0.0514 (9)	0.0389 (8)	−0.0028 (6)	0.0067 (6)	−0.0093 (6)
S2	0.0239 (3)	0.0230 (3)	0.0521 (4)	0.000	−0.0031 (3)	0.000
O21	0.0739 (12)	0.0411 (9)	0.0606 (11)	−0.0021 (8)	0.0280 (9)	−0.0121 (8)
O22	0.0993 (18)	0.142 (2)	0.0884 (16)	−0.0935 (18)	−0.0563 (14)	0.0677 (16)

### Geometric parameters (Å, º)

**Table:**

C1—C2	1.389 (3)	N1—S1	1.5950 (19)
C1—C6	1.390 (3)	N1—H1N	0.834 (17)
C1—S1	1.7887 (18)	N1—H2N	0.866 (17)
C2—C3	1.397 (3)	N2—H2A	0.8900
C2—H2	0.9300	N2—H2B	0.8900
C3—C4	1.379 (3)	N2—H2C	0.8900
C3—H3	0.9300	S1—O2	1.4279 (15)
C4—C5	1.386 (3)	S1—O1	1.4494 (15)
C4—N2	1.475 (2)	S2—O22^i^	1.4362 (19)
C5—C6	1.399 (3)	S2—O22	1.436 (2)
C5—H5	0.9300	S2—O21^i^	1.4750 (18)
C6—H6	0.9300	S2—O21	1.4751 (18)
			
C2—C1—C6	120.56 (17)	H1N—N1—H2N	117 (3)
C2—C1—S1	119.64 (14)	C4—N2—H2A	109.5
C6—C1—S1	119.80 (14)	C4—N2—H2B	109.5
C1—C2—C3	120.16 (17)	H2A—N2—H2B	109.5
C1—C2—H2	119.9	C4—N2—H2C	109.5
C3—C2—H2	119.9	H2A—N2—H2C	109.5
C4—C3—C2	119.00 (17)	H2B—N2—H2C	109.5
C4—C3—H3	120.5	O2—S1—O1	118.39 (10)
C2—C3—H3	120.5	O2—S1—N1	107.08 (10)
C3—C4—C5	121.45 (17)	O1—S1—N1	111.18 (11)
C3—C4—N2	118.95 (16)	O2—S1—C1	106.84 (9)
C5—C4—N2	119.59 (17)	O1—S1—C1	106.76 (9)
C4—C5—C6	119.60 (18)	N1—S1—C1	105.83 (10)
C4—C5—H5	120.2	O22^i^—S2—O22	107.4 (3)
C6—C5—H5	120.2	O22^i^—S2—O21^i^	109.11 (16)
C1—C6—C5	119.24 (17)	O22—S2—O21^i^	110.07 (12)
C1—C6—H6	120.4	O22^i^—S2—O21	110.07 (12)
C5—C6—H6	120.4	O22—S2—O21	109.11 (16)
S1—N1—H1N	117.4 (19)	O21^i^—S2—O21	111.04 (16)
S1—N1—H2N	121.8 (19)		
			
C6—C1—C2—C3	−0.4 (3)	S1—C1—C6—C5	−179.13 (16)
S1—C1—C2—C3	179.02 (16)	C4—C5—C6—C1	−0.1 (3)
C1—C2—C3—C4	0.3 (3)	C2—C1—S1—O2	21.96 (19)
C2—C3—C4—C5	−0.2 (3)	C6—C1—S1—O2	−158.59 (17)
C2—C3—C4—N2	178.55 (18)	C2—C1—S1—O1	149.55 (17)
C3—C4—C5—C6	0.1 (3)	C6—C1—S1—O1	−31.01 (19)
N2—C4—C5—C6	−178.65 (18)	C2—C1—S1—N1	−91.92 (18)
C2—C1—C6—C5	0.3 (3)	C6—C1—S1—N1	87.52 (18)

Symmetry code: (i) −*x*+1, *y*, −*z*+3/2.

### Hydrogen-bond geometry (Å, º)

**Table:**

*D*—H···*A*	*D*—H	H···*A*	*D*···*A*	*D*—H···*A*
N1—H1*N*···O22^i^	0.84 (2)	1.93 (2)	2.743 (3)	164 (3)
N1—H2*N*···O21^ii^	0.86 (2)	1.99 (2)	2.849 (3)	168 (3)
N2—H2*B*···O2^iii^	0.89	2.30	3.043 (3)	141
N2—H2*B*···O1^iv^	0.89	2.57	3.146 (2)	123
N2—H2*A*···O22^v^	0.89	1.90	2.787 (3)	177
N2—H2*C*···O21^vi^	0.89	2.06	2.900 (3)	158

Symmetry codes: (i) −*x*+1, *y*, −*z*+3/2; (ii) −*x*+1/2, *y*+1/2, *z*; (iii) −*x*, −*y*+1, −*z*+1; (iv) *x*, −*y*+1, *z*−1/2; (v) *x*−1/2, −*y*+3/2, −*z*+1; (vi) −*x*+1, −*y*+1, −*z*+1.

## References

[bb1] Bruker (2001). *SAINT* and *SMART* Bruker AXS Inc., Madison, Wisconsin, USA.

[bb2] Desiraju, G. R. & Steiner, T. (1999). *The Weak Hydrogen Bond in Structural Chemistry and Biology*, p. 13. International Union of Crystallography and Oxford Science Publications.

[bb3] Etter, M. C., MacDonald, J. C. & Bernstein, J. (1990). *Acta Cryst.* B**46**, 256–262. 10.1107/s0108768189012929 2344397

[bb4] Kent, M. (2000). *Advanced Biology*, p. 46. New York: Oxford University Press Inc.

[bb5] Nardelli, M. (1995). *J. Appl. Cryst.* **28**, 659.

[bb6] Pandiarajan, S., Balasubramanian, S., Ravikumar, B. & Athimoolam, S. (2011). *Acta Cryst.* E**67**, o2788. 10.1107/S1600536811038827 PMC3201237 22058822

[bb7] Petricek, V., Dusek, M. & Palatinus, L. (2006). *JANA2006* Institute of Physics of the Czech Academy of Sciences, Prague, Czech Republic.

[bb8] Schomaker, V. & Trueblood, K. N. (1968). *Acta Cryst.* B**24**, 63–76.

[bb9] Sheldrick, G. M. (2008). *Acta Cryst.* A**64**, 112–122. 10.1107/S0108767307043930 18156677

[bb10] Spek, A. L. (2009). *Acta Cryst.* D**65**, 148–155. 10.1107/S090744490804362X PMC2631630 19171970

[bb11] Zaouali Zgolli, D., Boughzala, H. & Driss, A. (2010). *Acta Cryst.* E**66**, o1488. 10.1107/S1600536810019471 PMC2979367 21579552

